# Long-term use of ultra-low-dose mifepristone for uterine leiomyoma control and safety discussion: A case report

**DOI:** 10.1097/MD.0000000000032413

**Published:** 2022-12-30

**Authors:** Guangdi Zhou, Qin Li

**Affiliations:** a MOE-Shanghai Key Laboratory of Children's Environmental Health, Xinhua Hospital, Affiliated to Shanghai Jiao Tong University School of Medicine, Shanghai, China; b Department of Obstetrics and Gynecology, Shanghai Changhai Hospital, China.

**Keywords:** dose mifepristone, long, low, term use, ultra, uterine leiomyoma

## Abstract

In recent years, many clinical trials have used low-dose mifepristone to control uterine fibroids, some of which have achieved good results. However, according to the classic concept, long-term use of mifepristone could induce malignant transformation of the endometrium, and the effect of ultra-low-dose of mifepristone on uterine fibroids is still uncertain. Researchers are short on cases of uterine fibroids treated with mifepristone for long periods (more than a year).

**Patient concerns:**

A 47-year-old woman went to the hospital because of anemia.

**Diagnoses:**

The patient was diagnosed with uterine leiomyoma.

**Interventions:**

The patient refused the suggestion of surgery, she was continuously treated with an ultra-low-dose (12.5 mg per 5 days) of mifepristone monotherapy for 4 years.

**Outcomes:**

The uterine leiomyoma was stable, anemia and other symptoms disappeared, and the menstrual cycle, liver and kidney function, and tumor markers were normal.

**Lessons:**

Judging from our case, long-term, ultra-low-dose mifepristone for uterine fibroids treatment was safe and more effective than conventional therapy.

## 1. Introduction

Uterine leiomyoma is one of the most common female reproductive system diseases. It frequently occurs in women of childbearing age from 30 to 50 years old. It can cause abnormal uterine bleeding, pain, and other symptoms and is the main reason for clinical hysterectomy, which greatly adversely impacts women’s reproductive health and fertility. The etiology of uterine leiomyoma has not been completely determined. The relatively clear inducements of uterine leiomyoma include Med12 gene mutation,^[1–4]^ mitochondrial mutation,^[5]^ p53 signal pathway abnormality,^[6]^ etc. Estrogen and progesterone can accelerate the growth of uterine leiomyoma.^[7]^ Progesterone receptors directly activated by progesterone can promote the proliferation of uterine leiomyoma, and selective progesterone receptor modulators can treat uterine leiomyoma.^[8,9]^

Mifepristone, also known as RU486, is a norethisterone derivative synthesized in the 1980s. It does not have progesterone, estrogen, and androgen activities but has a strong affinity with progesterone receptors, which can antagonize progesterone effects, terminate a pregnancy, and control uterine fibroids.^[10,11]^ According to the traditional concept, mifepristone should not be used for a long time because after mifepristone antagonizes progesterone, the endometrium is persistently stimulated by estrogen, which increases the risk of endometrial disease.^[12–14]^ This concept has been widely accepted and even included in the teaching book.^[15]^ However, it does not explain how long “the long time” is, although the general view is half a year. Therefore, the continuous use of mifepristone usually does not exceed half a year. We could not find publicly published cases involving the use of mifepristone for more than 1 year, and cases involving the use of mifepristone for many years and relevant data are lacking.

We report a case of multiple uterine fibroids that tend to increase in size. The maximum diameter was about 6.0 cm. The patient refused surgery and used an ultra low dose of mifepristone (12.5 mg per 5 days) to control the progression of uterine leiomyoma. After 4 years of monotherapy by single-drug mifepristone, the patient’s condition was stable, the fibroids sizes were not enlarged, symptoms such as anemia and menstrual dysfunction disappeared, and her menstrual cycle, blood routine, liver and kidney functions, and tumor markers were all normal.

## 2. Case presentation

**Figure 1.**
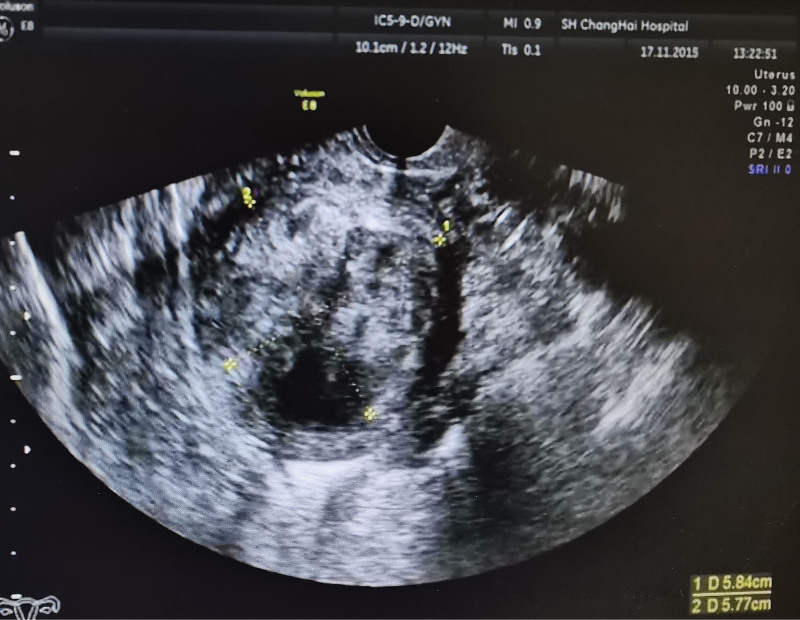
Transvaginal ultrasound at the patient's first visit in 2015. The diameter of uterine leiomyoma was about 5.8 cm.

The patient was born in 1968, married at 26, gave birth to a daughter naturally, and her husband and daughter were in good health. She had a regular menstrual cycle, moderate menstruation, and no obvious dysmenorrhea. At the age of 47 in 2015, uterine leiomyoma was found on physical examination, with a diameter of about 5.8 cm (Fig. 1), and left untreated. There was no obvious increase in uterine fibroids during follow-up. In February 2017, menorrhagia appeared, but she did not care about it. In February 2018, at 50, the uterine volume increased to the size of a 3-month-old pregnancy. Ultrasound reexamination revealed multiple uterine fibroids, with a maximum diameter of 6.0 cm, an endometrial thickness of 0.8 cm, and hemoglobin (HGB) of 80 g/L. The patient refused surgery and asked for drug treatment. The patient took 12.5 mg per day of mifepristone orally for 3 months, after which ultrasound reexamination revealed multiple uterine fibroids, with a diameter of 6.1 cm, an inner membrane thickness of 0.8 cm, the HGB increased to 141 g/L. The patient continued to take 12.5 mg per day of mifepristone for another 3 months. During this period, amenorrhea occurred. The uterine fibroids sizes had not changed, the endometrial thickness was 0.5 cm, and HGB was 140 g/L. Blood routine tests, liver and kidney functions were monitored, and no abnormalities were found.

**Figure 2.**
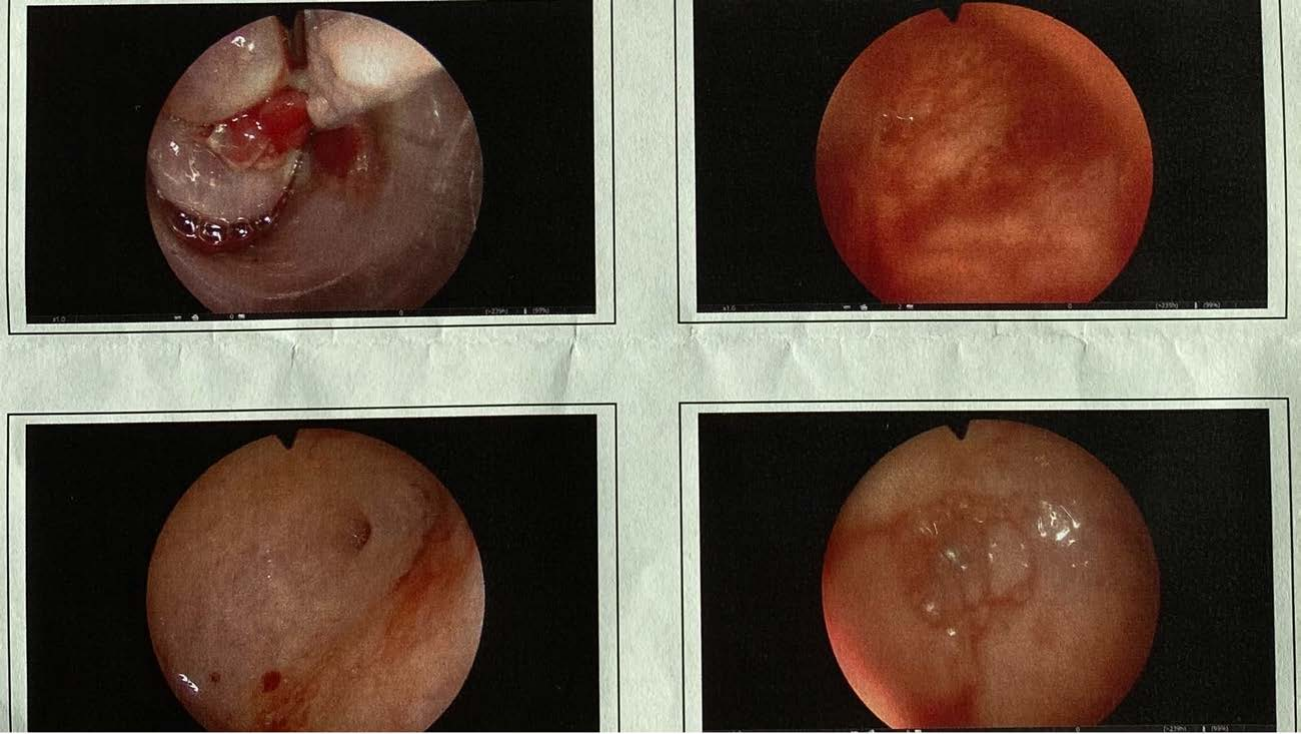
Hysteroscopic surgery figure of the patient due to uterine polyps in 2021, 3 and a half years after she taking low-dose mifepristone. Postoperative pathological examination showed that her endometrium was in good condition without malignant lesions.

Until August 2018, the patient was treated with mifepristone 12.5 mg per day for 6 months. Anemia and menorrhagia disappeared, but the uterine fibroids did not shrink. For fear of inducing malignant lesions of the uterus with continued long-term oral mifepristone, we suggested surgery and discontinuation of the mifepristone. She refused surgery and insisted on oral mifepristone but changed the dose to 12.5 mg per 5 days. Soon, her menstrual cycle resumed. The patient continued to use the mifepristone at a dose of 12.5 mg per 5 days until 2022. During these 4 years, her condition was stable, the myoma size did not increase, the menstrual cycle was normal, and her blood routine, liver and renal function, and tumor markers were normal. In August 2021, the patient underwent a hysteroscopic electroresection of the uterine polyp. The postoperative pathology was endometrial polyp (Fig. 2), and no malignant lesion was found. The patient experienced natural menopause in July 2022 and stopped mifepristone therapy in September 2022.

## 3. Discussion

Mifepristone is widely used to terminate pregnancies and treat endometriosis and ovarian and cervical cancers; it is also commonly used to treat uterine fibroids.^[15]^ Due to concerns about the potential of malignant changes in the uterine endometrium induced by the long-term use of mifepristone,^[14,16]^ researchers have tried to control uterine fibroids with lower doses. The recommended dose for treating uterine leiomyoma with mifepristone is 10 to 12.5 mg daily.^[16]^ Clinically common doses are 5 to 50 mg per day,^[17–27]^ whereas a few studies have reduced the dose of mifepristone to a minimum of 2.5 mg per day.^[28–30]^ The researchers reported that after a 6-month open-label trial, the 2.5 mg per day mifepristone dosage resulted in a lesser reduction in fibroid size but a similar improvement in quality of life compared to the 5 mg dose.^[28]^ The rate of amenorrhea was the highest after 2 and 3 months of treatment; afterwards, about half the women’s menstrual cycle gradually recovered. The 2.5 mg per day mifepristone dose was too low to maintain amenorrhea consistently. Endometrial sampling showed cystic glandular dilatation, but no endometrial hyperplasia or cellular atypia was found.

Lower doses of long-term mifepristone are used for contraception, and the most commonly used dose is 2.5 to 5 mg daily.^[31,32]^ Bygdeman attempted a clinical trial of 0.5 mg and 0.1 mg daily doses of mifepristone for 3 months.^[33]^ He found that 0.1 mg per day had no significant effect on the endometrium, while a dose of 0.5 mg per day caused a slight slowing of endometrial development and a significant reduction in glandular diameter. The immunoreactivity of estrogen and progesterone receptors did not change significantly. The results confirm that even a dose of mifepristone too low to interfere with ovulation can significantly affect the endometrium. All the clinical trials on mifepristone were conducted over 3 to 12 months; no longer-term drug trials were found.

These results have provided a theoretical basis for the case we report. Our patient was on single-drug low-dose mifepristone from age 50 until age 54. She successfully controlled the progression of uterine fibroids, her menstrual cycle remained normal, and symptoms such as anemia improved. This is consistent with previous findings that a daily dose of 2.5 mg rarely causes persistent amenorrhea but can significantly affect endometrial development and hormone receptor expression.^[28–30]^ An ultra-low dose of mifepristone can play an active role in the control of uterine fibroids.

Studies have shown that mifepristone at a dose of 50 mg daily for 6 months results in frequent mitotic figures in the endometrium with no cytological abnormalities.^[14]^ Endometrial glands were irregular in size and shape after several months of 5 mg per day or less mifepristone, and no cytologic atypia was seen.^[28,33,34]^ There is no evidence that ultra-low-dose (2.5 mg per day or less) mifepristone over a long period can cause endometrial malignant transformation, and our case proves this point. After several years of ultra-low-dose mifepristone, our patient underwent a hysteroscopic curettage biopsy, and no lesion was found in the endometrium. Our case provides confidence and reference for a longer period of low-dose mifepristone to control uterine fibroids.

## Acknowledgments

We would like to thank “The role and molecular mechanism of C20orf116 in ovarian cancer cells,” Changhai Hospital Youth Start-up Fund to support this study.

## Author contributions

Qin Li treated the patient, communicated with the patient, collated data, designed and planed this study. Guangdi Zhou wrote and revised this manuscript.

**Conceptualization:** Guangdi Zhou, Qin Li.

**Data curation:** Qin Li.

**Funding acquisition:** Qin Li.

**Investigation:** Qin Li.

**Writing – original draft:** Guangdi Zhou.

**Writing – review & editing:** Guangdi Zhou.
